# Genetic correlation analysis identifies genetic loci shared between Alzheimer’s disease and primary psychiatric disorders

**DOI:** 10.1002/alz.093421

**Published:** 2025-01-03

**Authors:** Ajneesh Kumar, Nicholas R. Ray, Michael L. Cuccaro, Gary Beecham, Edward D. Huey, Christiane Reitz

**Affiliations:** ^1^ Columbia University, New York, NY USA; ^2^ Columbia University Irving Medical Center, New York, NY USA; ^3^ John P. Hussman Institute for Human Genomics, University of Miami Miller School of Medicine, Miami, FL USA; ^4^ G. H. Sergievsky Center, Vagelos College of Physicians and Surgeons, Columbia University, New York, NY USA

## Abstract

**Background:**

Neuropsychiatric Symptoms (NPS) (e.g., aggression, psychosis, anxiety, apathy, depression, agitation, sleep disturbances, repetitive behaviors) occur in 85% of AD patients, and are associated with accelerated decline, out‐of‐home placement, increased costs, and greatly increased suffering of patients and families. Our understanding of the etiology of NPS in AD is inadequate, with treatments for NPS often being ineffective and associated with serious adverse effects. Pharmacological treatments have generally been borrowed from their indications for psychiatric illness, despite limited understanding to what extent the molecular and genetic architectures underlying AD‐associated NPS overlap with that of primary psychiatric disorders.

**Method:**

To characterize the genetic overlap between AD and major psychiatric disorders and identify potentially shared biological processes we conducted local genetic correlation analyses between AD and bipolar disorder, depression, and schizophrenia using LAVA (Local Analysis of [co]Variant Association), capitalizing on the largest most recent GWAS summary statistics for these traits (AD: n = 401,577; bipolar disorder: n = 413,466; depression: n = 1,154,267; schizophrenia: n = 130,644).

**Result:**

Local genetic correlation analyses identified fifteen loci shared between Alzheimer’s disease and depression and two loci shared between AD and bipolar disorder after adjusting for multiple testing. Finemapping and functional analyses of the identified loci are in process.

**Conclusion:**

These findings support the notion of genetic overlap between AD and bipolar disorder, and AD and depression, respectively. Functional characterization of these loci will be critical to characterize the specific underlying causative genes and biological processes.

